# Implementation of a psychomotor vigilance test to investigate the effects of driving fatigue on oil and gas truck drivers’ performance

**DOI:** 10.3389/fpubh.2023.1160317

**Published:** 2023-10-05

**Authors:** Al-Baraa Abdulrahman Al-Mekhlafi, Ahmad Shahrul Nizam Isha, Maged S. Al-Quraishi, Noreen Kanwal

**Affiliations:** ^1^Faculty of Leadership and Management, Universiti Sains Islam Malaysia (USIM), Nilai, Malaysia; ^2^Department of Management & Humanities, Universiti Teknologi PETRONAS, Seri Iskandar, Perak, Malaysia; ^3^Faculty of Engineering, Thamar University, Dhamar, Yemen; ^4^Centre for Digital Home, Faculty of Engineering, Multimedia University, Cyberjaya, Malaysia; ^5^Department of Management, Sunway Business School, Sunway University, Bandar Sunway, Malaysia

**Keywords:** driving fatigue, driving performance, truck drivers, psychomotor vigilance test, oil and gas transportation

## Abstract

**Introduction:**

Driving fatigue has been shown to increase the risk of accidents and potentially fatal crashes. Fatigue is a serious risk that some drivers do not take seriously. Previous studies investigated the effects of driving fatigue in the Malaysian oil and gas transportation industry by employing survey questionnaires. However, they did not explain the behavior of fatigue. Besides, these results required validation by a more reliable method that can describe how fatigue occurs.

**Methods:**

Thus, in this study, we used the Psychomotor Vigilance Test (PVT-192) and a short survey to address driving fatigue behavior and identify the influences of driving fatigue on driving performance in real life (on the road) with actual oil and gas tanker drivers. The total participants in the experimental study were 58 drivers.

**Results:**

For the analysis, a Wilcoxon Signed Ranks Test, Z value and Spearman’s rho were used to measure the significant difference between the pre and post-tests of PVT and the correlation between the fatigue variables and driving performance.

**Discussion:**

During the experiment’s first and second days, this study’s results indicated that driving fatigue gradually escalated. Likewise, there was a negative correlation based on the test of the relationship between the PVT data and the driving performance survey data. Additionally, the drivers suffer from accumulative fatigue, which requires more effort from the transportation company management to promote the drivers awareness of fatigue consequences.

## 1. Introduction

Each year, over 1.3 million individuals are killed in vehicle crashes. Despite owning 60% of the world’s vehicles, low- and middle-income countries are responsible for 93% of road fatalities. Most nations lose 3% of their GDP due to traffic accidents (1). It is believed that fatigue contributes to 10 to 20% of all traffic accidents worldwide (2). According to the National Highway Traffic Safety Administration, around 100,000 police-reported drowsy-driving crashes occur yearly in the United States, resulting in roughly 800 fatalities and approximately 50,000 injuries (3). As per official data from the United States, truck drivers are more prone to fatigue than other drivers in the 120,000 nationwide fatal and catastrophic traffic accidents, with driving fatigue accounting for 13% of truck incidents (4). In Malaysia, the Malaysian Institute of Road Safety Research (MIROS) found that fatigue is one of the leading causes of vehicle, truck, and bus accidents (5). In addition, another MIROS research of commercial truck drivers in Klang Valley found that 17.7% of them were fatigued (6).

Driving fatigue is commonly described as a lack of adequate sleep or a feeling of sleepiness that can reduce alertness and concentration, making drivers less able to notice incoming risks (7). Fatigue is a condition that decreases physical or mental attentiveness, hindering the performance of cognitive and psychomotor tasks (8, 9). Fatigue impairs physiological and psychological performance by slowing sensorimotor activities and impairing information processing (10). As a result, it may reduce the ability of the driver to respond effectively to an unusual, unexpected, or emergency circumstance (7, 11). Fatigued drivers have slower response times and less attention, awareness, and control of their vehicles. According to research, driving fatigued is just as risky as driving drunk (12, 13). According to Zhang (14), the driver’s effective response time tends to be prolonged because of drowsiness or fatigue.

For several years, Malaysian researchers have been focusing on driving fatigue in an attempt to understand how fatigue affects drivers’ tasks among drivers of Malaysian oil and gas tankers. According to Sabir (15), fatigue is potentially dangerous for the energy transportation sector. Thus, fatigue risk factors must be countered to prevent vehicle crashes. Likewise, in their survey study, Sabir et al. (16) found that fatigue mediates the relationship between perceived stress and aberrant driving behavior of oil and gas truck drivers. Moreover, Krishnan et al. (17) indicated a significant relationship between psychological risk factors and fatigue among drivers of oil and gas tankers. Besides, in their survey study, Al-Mekhlafi et al. (18) demonstrated that work schedules and activities lead to fatigue which will impact driving performance (19). However, previously published studies are limited to a survey approach. In their limitations, Krishnan et al. (17) state that there is a need to incorporate another method besides questionnaires among oil and gas drivers into the methodology to achieve valid and reliable results. Furthermore, it is necessary to use a particular fatigue screening instrument for Malaysian oil and gas tanker drivers (16).

It can be concluded from the above previous studies there is a method gap that requires to be addressed within the context of the energy transportation industry. At the same time, there is a lack of studies that explain how fatigue impacts driving performance among drivers of oil and gas tankers. Consequently, the current study filled this gap by investigating how driving fatigue impacts the driving performance of Malaysian drivers working in the energy industry via the Psychomotor Vigilance Test (PVT-192). This study conducted this experiment using PVT-192 in Malaysia’s actual tankers parking of energy transportation companies, and the drivers have tested in pre and post of their tasks. As a result, the study aims to investigate how driving fatigue affects the driving performance of Malaysian oil and gas tanker drivers. In addition, the study aims to discover what kind of fatigue drivers suffer from.

## 2. Methodology

**Figure 1:**
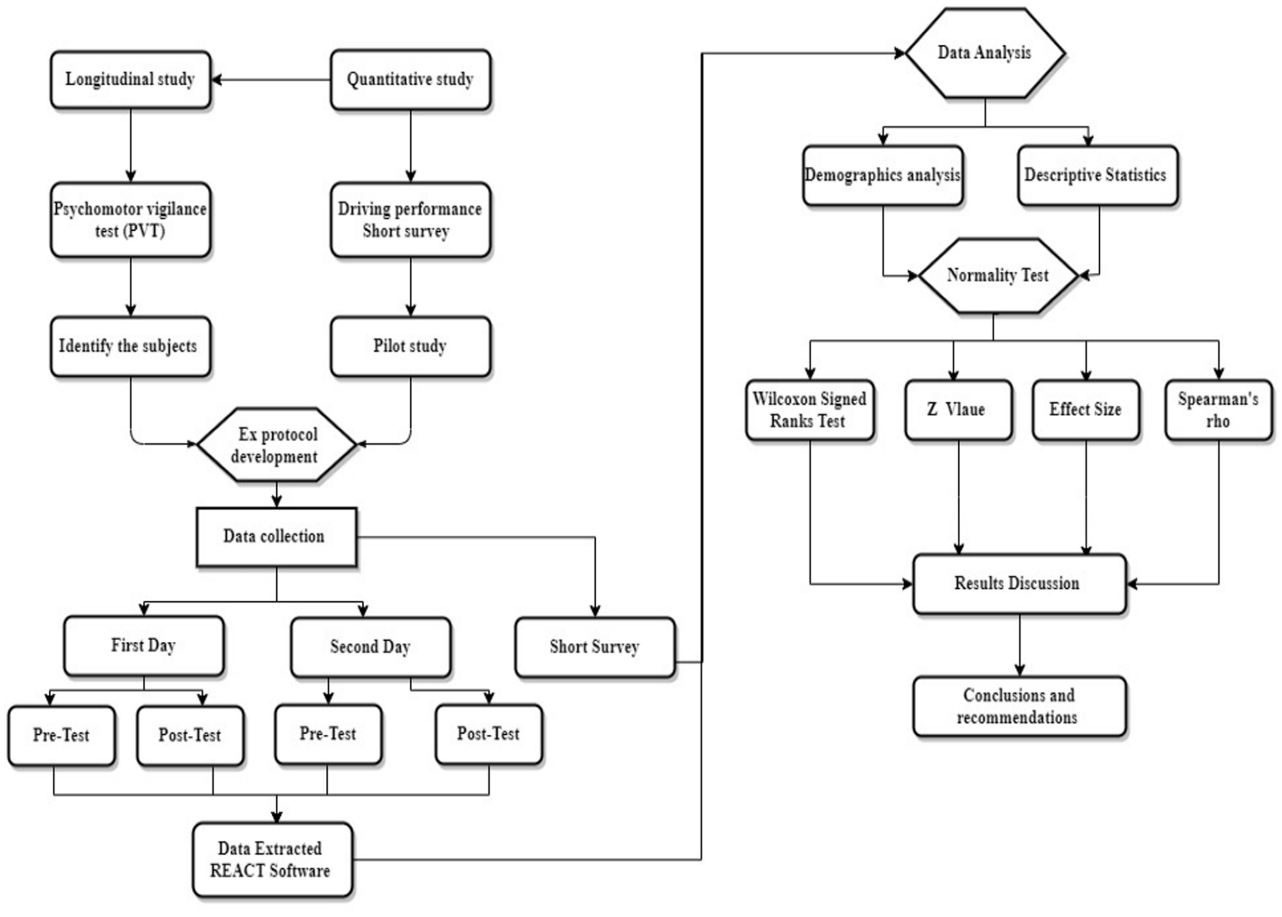
Research design.

The research design is presented in Figure 1. The study employed a quantitative research approach, utilizing the psychomotor vigilance test (PVT) (reaction time and Lapses RT > 500 ms) and a short driving performance questionnaire. The study protocol was developed based on a pilot study and subject identification. The PVT tests were conducted over two experimental days, with each subject completing four tests (two before driving and two after). Subsequently, the data was extracted from the PVT device using REACT software. During the analysis stage, the collected data from PVT and the short questionnaire were subjected to several tests. Firstly, descriptive analysis was conducted on the variables and demographic information. Secondly, a normality test was carried out, revealing that the data was not normally distributed (MeanRT = value of p 0.046, time, Lapses = value of *p* <0.001). Therefore, non-parametric tests, such as the Wilcoxon Signed Ranks Test, Z values, and Spearman’s rho, were employed to test for significant differences and correlations.

### 2.1. Sampling design technique

This study used a purposive sampling technique to design the sample for the fatigue assessment test. The sample size for the experiment was determined based on the mean sample size of previous studies that used the PVT 3–5 min in the last three years (20–25). The minimum sample size for this study will be 40 based on the mean of the largest (72) and smallest (16) sample sizes.

However, the sample size was calculated using the G*Power analysis software (26). Utilizing the G*Power software, a minimum sample size of *n*=41 was determined, considering a power (1-β) of 0.95, an effect size of 0.5, and an alpha level of 0.05. Consequently, this study gathered data from a sample size exceeding the calculated minimum. The final participant count in this study comprised 44 subjects (*n*=44). As a result, the sampling power of the study stands at 0.95, surpassing the recommended minimum criterion of 0.8 (27).

### 2.2. Participants criteria

Eligibility criteria included: (1) age 21–60; (2) owning a valid heavy vehicle driver’s license; (3) long-haul drivers who just returned from the weekend break; and (4) who can read and write. The study was conducted in the oil and gas transportation industry in different states of Malaysia. The total participants in the experimental study were 58 oil and gas tanker drivers. However, only 44 subjects completed 2 days and four tests, so we eliminated incomplete data from 14 participants.

The pilot study was conducted before the main data collection stage to test the validity of PVT through ten oil and gas tanker drivers. Oil and gas tanker drivers were measured during both night and day shifts. The objective was to test the validity of PVT, determine if the 10-min test was appropriate for drivers, and identify any challenges to using PVT among drivers. As a result, the test time was reduced from 10 to 3 min in the main study, and then the protocol was developed.

### 2.3. Psychomotor vigilance test (PVT-192)

The psychomotor vigilance test is an electronic computerized test presentation utilized to assess alertness and personal behavior by visual reaction time (28). The PVT is a sustained attention and response time test that assesses how quickly a participant reacts to a visual stimulus. The test is well-known for its convergent validity and simple metrics (28).

The current study used the PVT-192 to measure driver fatigue over 2 days. Based on recommendations from the pilot study, the test time was reduced from 10 min to 3 min to suit the drivers’ time constraints better. The PVT-192 is a vigilance test that lasts for 3 min and measures response time to stimuli that appear randomly throughout the trial (29–31). A small screen displaying red numbers (the stimulus) is located on the front face and at the top of the device. Below the screen is another screen for setting up the test and entering subject information and trial numbers. Two square buttons are placed near the bottom of the handheld unit, and participants press the button on the side corresponding to their dominant hand in response to the red numbers displayed on the small screen at the top of the device. Right-hand participants should press the button on the right, while left-hand participants should press the button on the left.

The small red numbers indicated the reaction time of the participants in milliseconds. Participants were instructed to press the appropriate button when red numbers appeared on the small screen at the top of the device. Unclicking the button within 500 milliseconds of the presentation of the stimulus would result in a lapse (i.e., an omission error). PVT-192 recorded the mean reaction time (Mean RT) along with lapses.

The PVT offers several advantages, including its time efficiency and lack of training requirements. Only a brief description of the procedure is needed prior to administering the test. Moreover, compared to tools such as the Epworth Sleepiness Scale (ESS), which relies on subjective self-assessment, the PVT provides a more objective assessment of alertness (28). However, the PVT test also has its limitations. It may not be suitable for individuals with certain characteristics, such as color blindness, cognitive impairment, or physical impairments like Parkinson’s disease (32). These individuals may not be able to work as tanker drivers. Furthermore, given that most drivers have a low level of education, the PVT is appropriate for testing individuals with any level of education.

The evidence suggests that assessing lapses through measures such as reaction times and the number of lapses in tasks like the PVT can provide valuable insights into an individual’s level of fatigue. Understanding the relationship between fatigue and performance lapses can better identify and mitigate the risks associated with impaired task performance, including driving-related accidents.

### 2.4. Data collection procedures

**Figure 2:**
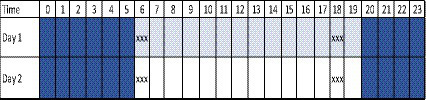
Protocol of fatigue assessment test.

This study focused on driving fatigue indicators, specifically mean RT and lapses, as measured by PVT tests conducted over 2 days. Eligible participants were monitored for their level of driver’s vigilance before and after driving. Participants were familiarized with the PVT test and given a 1-min PVT test as a trial, followed by a 3-min PVT test. Two tests were conducted each day - one before leaving the company parking lot as a pre-test and one upon returning to the parking lot as a post-test after actual driving. Drivers typically spend 12 h on one trip of daily driving based on their shift time unless there are obstacles on the road. In the fatigue assessment test protocol provided in Figure 2, the blue color indicates the time for sleeping, and the three X’s indicate the sequence of PVT test/driving/PVT test.

Participants completed a questionnaire about participant demographic information and driving performance as the dependent variable. This survey was collected one time at the end of the second day. The questionnaire items were adopted from Al-Mekhlafi et al. (33). Questionnaire items are “Operating entertainment systems do not distract me from driving (e.g., playing radio),”"Operating navigation systems do not distract me from driving.” “My reactions are faster than they used to be (e.g., braking in an emergency),”” I sometimes cannot judge my speed.” “I sometimes cannot hear the horns of other vehicles/sirens from emergency vehicles,” and “Sometimes my speedometer is hard to read during the daytime.” The respondents were required to answer each item based on a five-point Likert scale ranging from 1 = (Never) to 5 = (Always).

### 2.5. Ethical aspects

The Department of Management & Humanities approved the conduct of this study at Universiti Teknologi PETRONAS (YUTP-015LC0-043). As instructed, a brief introduction and training session were conducted to inform participants of the study’s objectives and to ask them to participate as volunteers. The participants provided their written informed consent to participate in this study.

### 2.6. Data analysis

Each response time (RT) value is stored in the device and later transferred to a PC for post-processing. The individual RTs were then analyzed to obtain summary statistics, such as the mean RT or the number of lapses (RTs > 500 ms) per session, using the REACT software (Ambulatory Monitoring, Inc., Ardsley, NY).

SPSS v25 was used to measure the significant difference between the PVT pre-test and post-test and the correlation between driving performance as the dependent variable and fatigue indicators (mean RT and lapses) as independent variables. To do a correlation test, first, we integrated the PVT and questionnaire data into one sheet to test the correlation between driving fatigue indicators (MRT and lapses) and driving performance. Second, classification has been done for PVT data to be appropriate for further analysis with questionnaire data; reclassification was from 1 to 5. A correlation test was calculated to indicate the relationship between the variables, driving performance as the dependent variable and fatigue indicators (mean RT, Lapses) as independent variables. Data analyses were conducted using SPSS v25 Non-parametric tests.

In this study, the 3-min PVT test was used to calculate PVT mean RT and lapses, which serve as indicators of driving fatigue and specify how reaction times increase, decrease, or stay the same over the 3-min test. A negative PVT mean RT slope implies that reaction times increase during the 3-min test, which worsens reaction time during the entire mission. A positive PVT mean RT slope reveals that reaction times have decreased during the 3-min test, which improves reaction time during the task. Meanwhile, a PVT mean RT slope of 0 shows no change in reaction time during the trial, indicating that reaction time neither improves nor worsens.

## 3. Results

### 3.1. Demographics analysis

Descriptive statistics for demographic information for the participants, such as the driver’s age and driving experience, are illustrated in Table 1.

**Table 1:** Demographic analysis of oil and gas tanker drivers.

Variable	Category	*n*	Percentage
Age	20–29 years old	10	22.7%
	30–39 years old	21	47.7%
	40–49 years old	11	25%
	50–59 years old	2	4.5%
Driver experience	Below than 1 year	7	15.9%
	1 year – 3 years	19	43.2%
	4 years – 6 years	10	22.7%
	7 years – 9 years	5	11.4%
	10 years – above	3	6.8%

### 3.2. Descriptive statistics of variables

The outcome variables in the descriptive statistics of PVT variables (mean RT, lapses RT > 500 ms) and driving performance. In the self-report of driving performance as the dependent variable for the study, the mean was 4.25, and the standard deviation was 0.35. For the independent variable PVT variables (mean reaction time and lapses), the mean of the Mean RT was 286.82 on the pre-test of the first day and 307.95 on the post-test of the first day. While in the second day, the mean of MeanRT was 306.69 in the pre-test, and the mean was 353.57 in the post-test. Thus, notably, the mean RT was increased gradually. Likewise, in terms of the Lapses measured, the mean was greater than the study criteria mean > 500 ms. In the pre-test of the first day, the mean was 1.09, while in the post-test, the mean was 2.11. On the second day of the experiment, the mean of lapses in the pre-test was 2.48, and the mean was increased to 3.02 in the post-test, as shown in Table 2.

**Table 2:** Descriptive statistics of PVT fatigue indicators of oil and gas tanker drivers.

Experimental days	PVT tests	*n*	Mean	Std. Deviation	Minimum	Maximum
First day	meanRT1a	44	286.82	63.93	194.00	427.00
	meanRT1b	44	307.95	84.25	210.00	565.00
Second day	meanRT2a	44	306.69	68.16	218.00	454.00
	meamRT2b	44	353.57	115.29	227.00	909.00
First day	Lapses1a	44	1.09	1.491	0	5
	Lapses1b	44	2.11	2.345	0	10
Second day	Lapses2a	44	2.48	1.486	1	7
	Lapses2b	44	3.02	1.759	1	8
Once	Driving performance	44	4.2545	0.34698	3.64	5.00

1a. Pre-test of first day, 1b. Post test for first day, 2a. Pre-test of second day, 2b. post test of second day.

### 3.3. Comparison of two consecutive days

As seen in Table 3, the results indicated A Wilcoxon Signed-Ranks test indicated that the meanRT of the post-test of PVT on the first day (mean rank = 22.50) was rated more favorably than the PVT pre-test with (mean rank = 21.78). In comparison, the meanRT of the post-PVT test on the second day (mean rank = 23.04) was rated more favorably than the PVT pre-test with (mean rank = 22.27).

**Table 3:** Significant different of PVT tests Mean RT of oil and gas tanker drivers.

Pvt tests	Ranks	*N*	Mean rank	Sum of ranks
meanRT1a - meanRT1b	Negative Ranks	30^a^	21.78	653.50
	Positive Ranks	13^b^	22.50	292.50
	Ties	1^c^		
	Total	44		
meanRT2a - meamRT2b	Negative Ranks	31^d^	22.27	690.50
	Positive Ranks	13^e^	23.04	299.50
	Ties	0^f^		
	Total	44		

Mean RT. Mean reaction time of responses.

a meanRT1a < meanRT1b.

b meanRT1a > meanRT1b.

c meanRT1a = meanRT1b.

d meanRT2a < meamRT2b.

e meanRT2a > meamRT2b.

f meanRT2a = meamRT2b.

In addition, Table 4 shows the Z value on the first day of the experiment was Z = −2.18, *p* = 0.029. Besides, the Z value of the second day was Z = −2.28, *p* = 0.023. Consequently, there is a significant difference between the MeanRT of the PVT tests in the pre and post-test and the first and second days. Thus, the mean RT was higher in the post-tests and the second day of the experiment.

**Table 4:** Determine the significance of the difference in MeanRT of oil and gas tanker drivers.

Test statistics	meanRT1a - meanRT1b	meanRT2a - meamRT2b
Z	−2.18^b^	−2.28^b^
Asymp. Sig. (2-tailed)	0.029	0.023

aWilcoxon Signed Ranks Test.

b Based on positive ranks.

In terms of the Lapses numbers, the Wilcoxon Signed-Ranks test indicated that the number of Lapses in the post-PVT test on the first day of experimental (mean rank = 18.25), which was rated more favorable than the number of Lapses in the PVT pre-test with (mean rank = 16.60). While in the second day of the experiment, the number of Lapses in the post-PVT test (mean rank = 13.29) was rated more slightly than the PVT pre-test (mean rank = 12.89), as seen in Table 5.

**Table 5:** Significant different of PVT tests Lapses >500 ms oil and gas tanker drivers.

Pvt tests	Ranks	*N*	Mean rank	Sum of ranks
Lapses1a - Lapses1b	Negative Ranks	25^a^	16.60	415.00
	Positive Ranks	8^b^	18.25	146.00
	Ties	11^c^		
	Total	44		
Lapses2a - Lapses2b	Negative Ranks	18^d^	12.89	232.00
	Positive Ranks	7^e^	13.29	93.00
	Ties	19^f^		
	Total	44		

a Lapses1a < Lapses1b.

b Lapses1a > Lapses1b.

c Lapses1a = Lapses1b.

d Lapses2a < Lapses2b.

e Lapses2a > Lapses2b.

f Lapses2a = Lapses2b.

Likewise, Table 6 shows the Z value of the number of lapses for the first day of the experiment was Z = −2.43, *p* = 0.015. However, the Z value of the number of lapses on the second day was Z = −1.90, *p* = 0.059. In comparison, on the second day of the experiment, the difference between the number of Lpases was insignificant between the pre and post of PVT tests. In contrast, there was a significant difference between the number of lapses on the first day of the experiment. Overall, the number of lapses in the post-tests was greater.

**Table 6:** Determine the significance of the difference of lapses >500 ms.

Test statistics	Lapses1a - Lapses1b	Lapses2a - Lapses2b
Z	−2.43^b^	−1.90^b^
Asymp. Sig. (2-tailed)	0.015	0.059


aWilcoxon Signed Ranks Test.

b Based on positive ranks.

**Figure 3:**
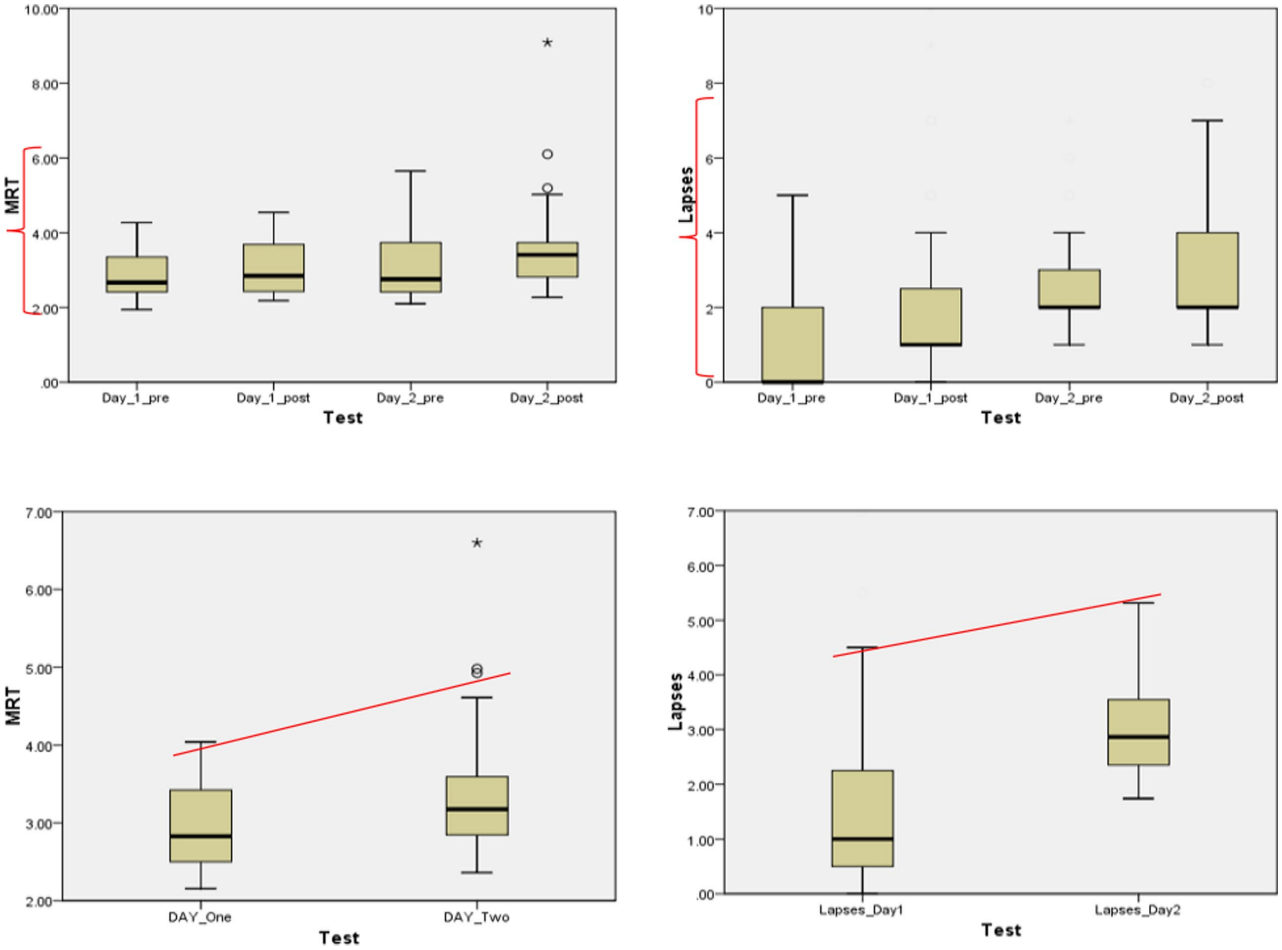
Comparisons of the mean reaction time (MRT) and lapses of the drivers during the two days of the experiment.

In comparison between the days of the PVT test, the results indicate that the impairment of driver’s performance appeared on the second day of the fatigue assessment test, which means the mean reaction time of drivers and the number of lapses in total was high on the second day as shown in Figure 3. The findings reveal a significant disparity between drivers’ performance on different days of the test; as seen in the figure, the performance dressed gradually, increasing driving fatigue.

Thus, it becomes evident that the most substantial decline in driver performance occurred on the post-tests and the second day of the fatigue assessment test.

Thus, after implementing the formula on the given results of the Wilcoxon Signed Ranks Test, the effect size of driving fatigue change is illustrated in Table 6. Based on Cohen (34), standards state that f^2^ ≥ 0.02, f^2^ ≥ 0.15, and f^2^ ≥ 0.35 reflect small, medium, and high impact sizes, respectively.

In order to evaluate if there were any changes in driving fatigue as a result of the delay in the reaction time on the stimulus of PVT pre and post-tests on the first day of the experimental, the Wilcoxon Signed Rank Test revealed a statistically significant positive change in driving fatigue as meanRT1a - meanRT1b Z = −2.180, with medium effect size (*r* = 0.23). The Lapses1a - Lapses1b Z = −2.430, with medium effect size (*r* = 0.25). Likewise, on the second day of the PVT test experimental, the Wilcoxon Signed Rank Test revealed a statistically significant positive change in driving fatigue as meanRT2a - meamRT2b Z = −2.282, with medium effect size (*r* = 0.24). However, the value of p for the second day’s Lapses2a - Lapses2b tests was not significant *p* = 0.059, but the Lapses2a - Lapses2b tests Z = −1.904, with medium effect size (*r* = 0.20) as shown in Table 7.

**Table 7:** Effect size of fatigue.

PVT tests		Z	*N*	Effect size	Statues
First day	meanRT1a - meanRT1b	–2.18	88	0.23	Medium
Second day	meanRT2a - meamRT2b	–2.28	88	0.24	Medium
First day	Lapses1a - Lapses1b	–2.43	88	0.25	Medium
Second day	Lapses2a - Lapses2b	–1.90	88	0.20	Medium

### 3.4. Correlation result analysis

Spearman’s rank correlation coefficient, often known as Spearman’s rho, is a mathematical formula used to calculate the strength and direction of a monotonic connection between two variables (35). Considering the outcomes, the PVT variables (Mean RT, Lapses) were negatively and significantly correlated with driving performance (*r* = −0.331, *p* = 0.028). It is worthwhile that the criteria of meaningful statistics are considered when *p* < 0.05 satisfies. It can be concluded that when the PVT variables (Mean RT, Lapses RT > 500 ms) level increases, driving performance will decrease (36), as found in Table 8.

**Table 8:** Correlation test.

Non-parametric correlations test		driving_performance	MeanRT_Lapses
driving_performance	Correlation Coefficient	1.000	−0.331^*^
	Sig. (2-tailed)	.	0.028
	N	44	44
MeanRT_Lapses	Correlation Coefficient	−0.331^*^	1.000
	Sig. (2-tailed)	0.028	.
	N	44	44

*Correlation is significant at the 0.05 level (2-tailed).

### 3.5. PVT3-Minute test performance over 2 days in detail

**Figure 4:**
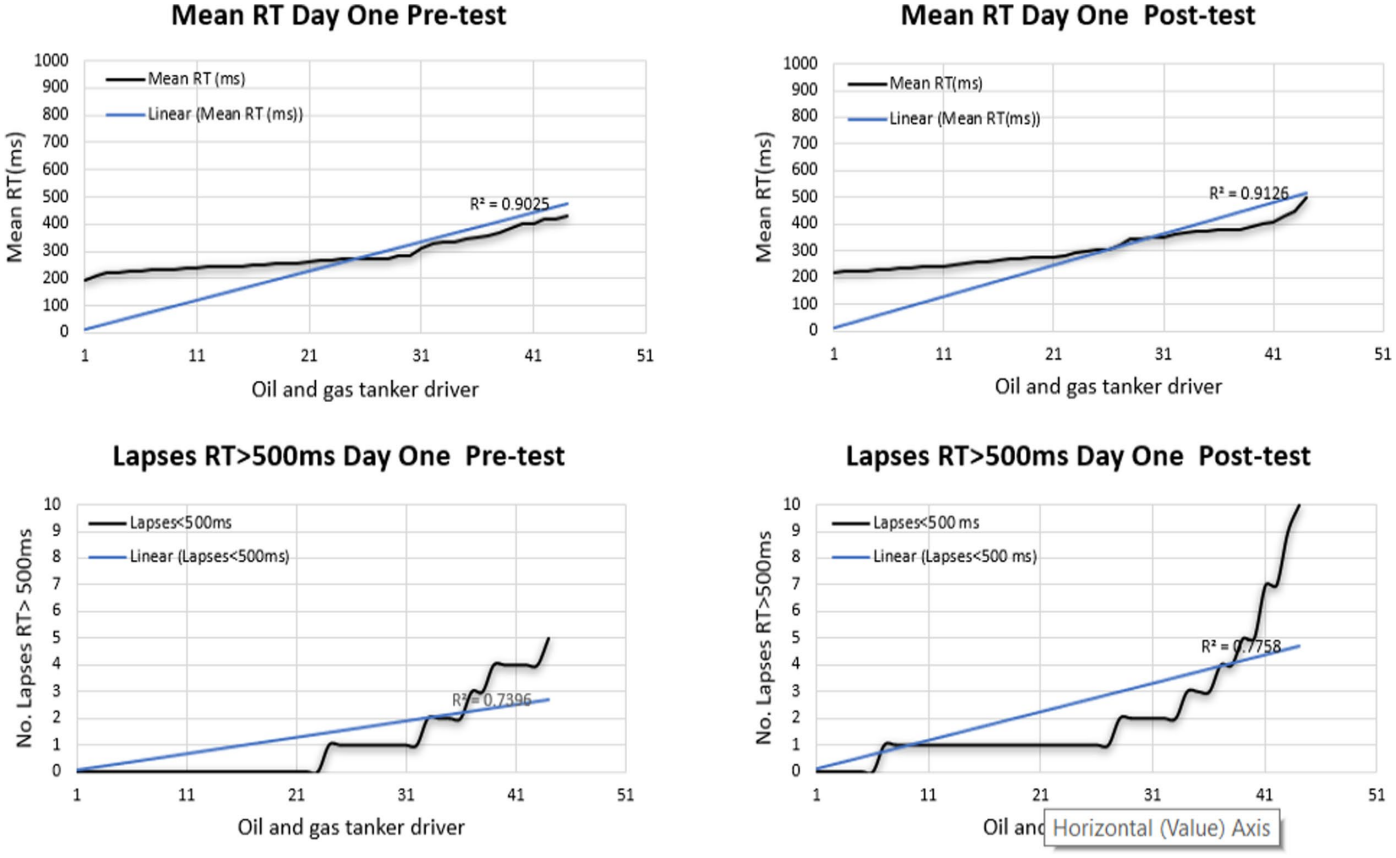
Performance of PVT test in the first-day pre and post.

**Figure 5:**
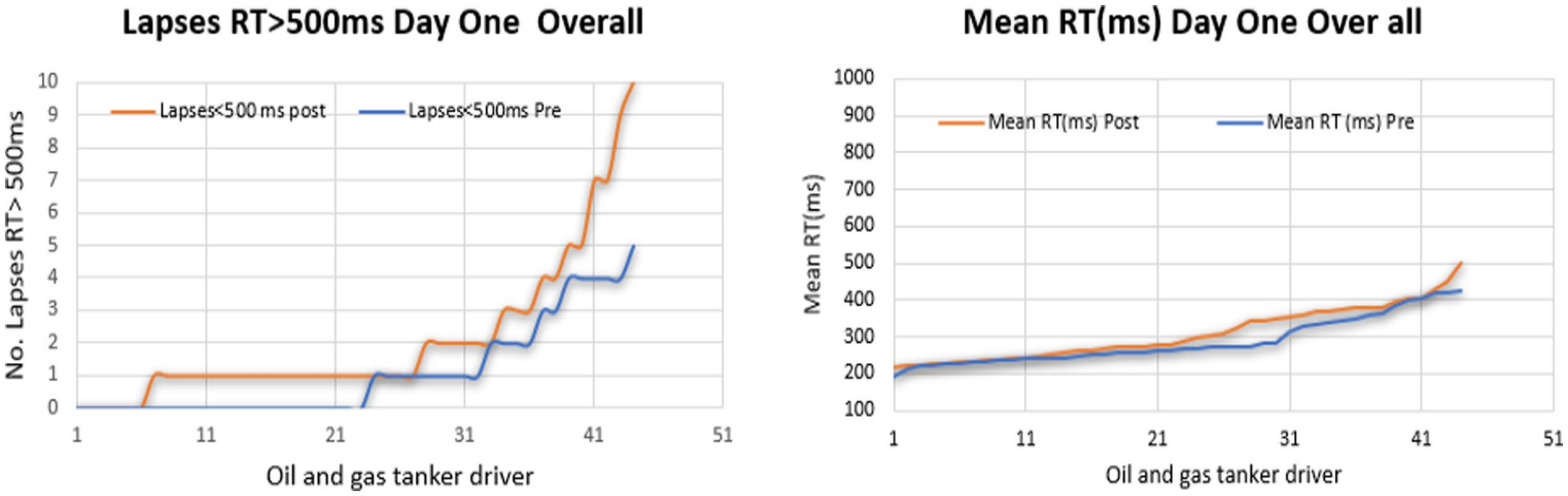
Performance of PVT test on the first day.

The line graphs illustrate the proportion of driver fatigue among oil and gas tanker drivers who underwent fatigue monitoring through the PVT test for 2 days. On the first day, a significantly greater percentage of lapses was observed in the post-test of the PVT test. Meanwhile, the highest percentage of MeanRT was observed in the post-test, as shown in Figures 4, 5.

**Figure 6:**
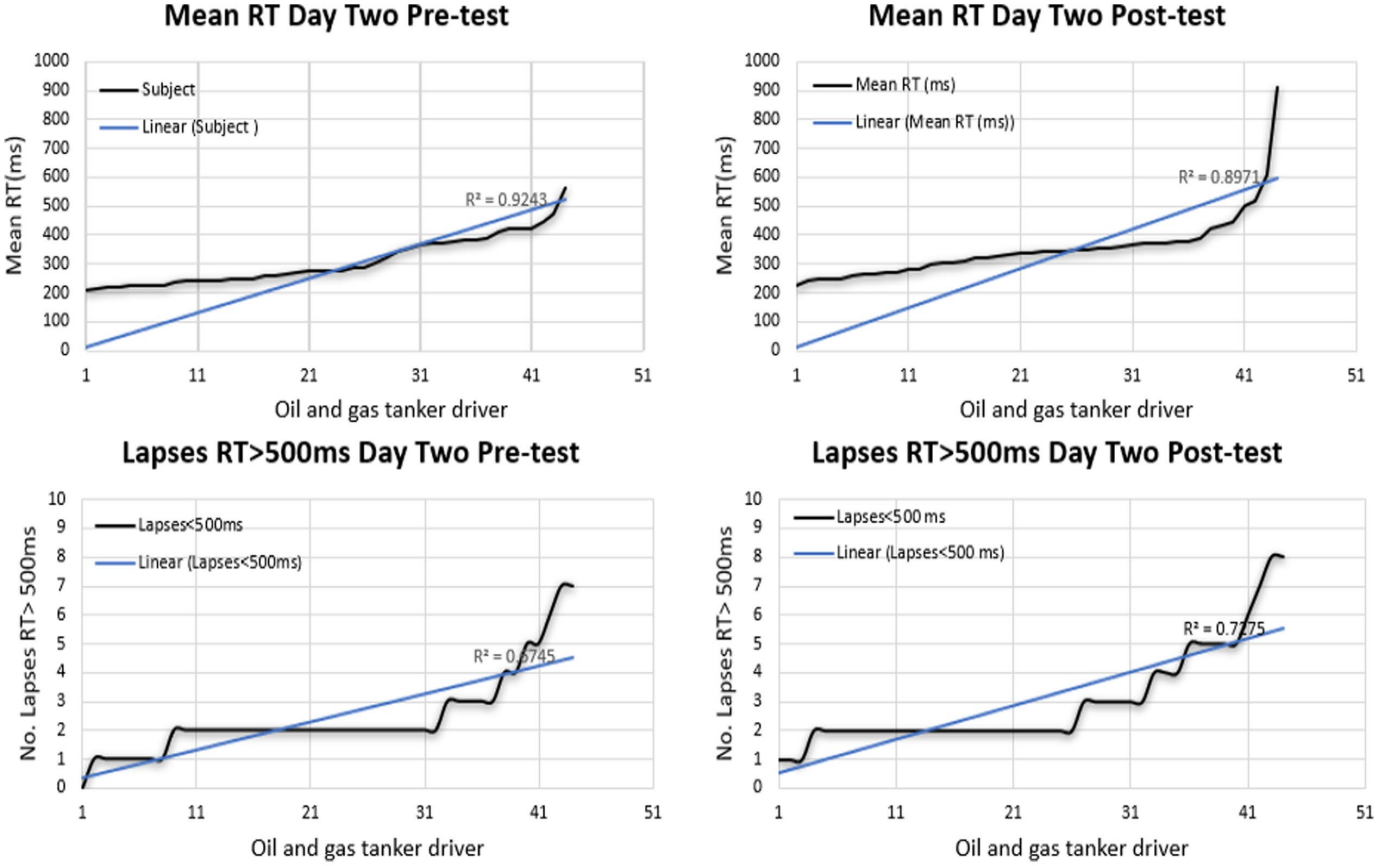
Performance of PVT test in the second-day pre and post.

**Figure 7:**
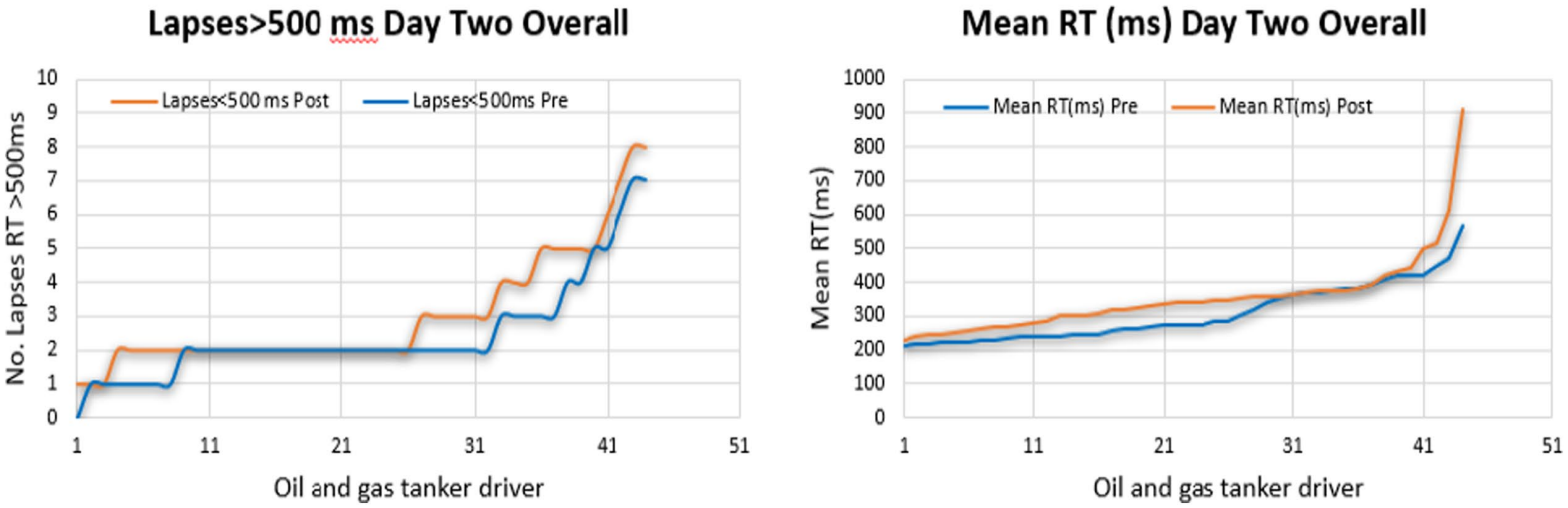
Performance of PVT test on the second day overall.

On the other hand, on the second day, the PVT test showed a greater percentage in the post-test of MeanRT indicators. The number of lapses showed a high level in the post-test, as seen in Figures 6, 7. Overall, fatigue was apparent in the post-test compared to the test’s pre-test time. Besides, fatigue was more obvious on the second day than on the first. Notably, sequential driving can lead to fatigue.

## 4. Discussion

Based on the results, this work has uncovered valuable insights into the impact of driving fatigue on driving performance. Fatigue gradually increases among oil and gas tanker drivers, indicating that Malaysian oil and gas tanker drivers experience cumulative fatigue. This accumulated fatigue often arises from extended periods of stress without sufficient recovery, leading to reduced productivity, compromised immune function, and decreased alertness. Additionally, the study has provided a clear illustration of how fatigue negatively affects DRIVING performance, as evidenced by testing the same subjects among drivers of oil and gas tankers.

Due to long travel distances and delivery time demands imparted on drivers of oil and gas tankers, the multitasking activities of drivers may be subject to the self-regulation of maintaining driver vigilance (37, 38). The role of vigilance in the creation time of drivers is still yet to be fully examined, particularly in the oil and gas driving context. The PVT test may be utilized to identify oil and gas tanker drivers who may be more susceptible to the negative effects of the monotonous road or drive for a long time. This study employed the PVT test four times two days before and after the driving tasks. The findings indicated a significant difference between the four tests of the PVT (Z = −2.18, −2.28, −2.43, −1.90).

This study also confirmed a significant influence of driving fatigue on driving performance based on PVT variables results. The results show a significant relationship between the Mean RT-Lapses and driving performance (r = −0.331, *p* = 0.028). It is worth noting that the criteria of meaningful statistics are considered when *p* < 0.05 is satisfied. Thus, the hypothesis for this relationship is supported; as PVT variables (Mean RT, Lapses RT > 500 ms) level increases, driving performance will decrease. This outcome is consistent with the existing literature (39–46).

In the transportation sector, fatigue occurs due to the nature of the driving job involving environmental interaction and shifting activities (41, 42). Mahachandra and Sutalaksana (47) demonstrated that shift frequency has consequences for the severity of poor vigilance and attention of drivers. Fatigue occurs when physiological activities increase, influencing drivers’ performance (48). Over time, fatigue has worsened, lowering alertness and productivity. For instance, fatigued drivers displayed less braking and frequently drove more than 10% above the speed limit during speed restriction parts. On the other hand, several fatigued drivers suffer from decreased decision-making capability and are likely to use braking systems unnecessarily, increasing fuel utilization (49).

Driving fatigue can significantly impact driving performance, and mitigating it can lead to improvements in overall driving performance. Several ways exist to mitigate driving fatigue and enhance driving performance. First, fatigue can hinder a driver’s ability to anticipate hazards on the road, such as other vehicles or pedestrians. By mitigating driving fatigue, drivers can maintain focus and improve their hazard anticipation skills (50). For example, a simulator experiment demonstrated that the SAFE-T training program improved hazard anticipation, mitigation, and attention maintenance skills among drivers who had been awake for 12 h (51). Second, fatigue can impair a driver’s response to hazards when they occur. Mitigating driving fatigue can enable drivers to respond faster and more accurately to road hazards (50, 51). Finally, fatigue can cause drivers to become less alert to their surroundings, thereby increasing the risk of accidents. By addressing driving fatigue, drivers can enhance their driving performance, ensuring they remain alert and attentive while driving (52).

The study found a significant relationship between MeanRT, Lapses and driving performance. As the level of PVT variables increased, driving performance decreased. This finding is consistent with existing literature in different fields. The study contributes to identifying drivers who may be more susceptible to the negative effects of monotonous road conditions or long hours of driving. By utilizing the PVT test, the study provides a practical method for assessing driver vigilance and identifying those who may require additional support or training to maintain alertness on the road. Likewise, the study emphasizes the importance of addressing the issue of driving fatigue in the transportation sector, particularly in industries such as oil and gas transportation, where long travel distances and delivery time demands are often imposed on drivers. The study’s findings provide evidence for the negative impact of fatigue on driving performance, which can lead to safety risks for drivers and other road users. Besides, the study highlights the need for strategies and interventions to reduce driving fatigue and promote driver vigilance, such as implementing regular breaks, providing training on fatigue management, and using technology to monitor driver alertness. By addressing the issue of driving fatigue, we can improve road safety and productivity, benefitting both drivers and society as a whole. Overall, the study underlines the significance of prioritizing driver safety and well-being in the oil and gas transportation industry and the need for continuous efforts to improve driver vigilance and reduce the risk of accidents caused by driving fatigue. By addressing the issue of driving fatigue, we can improve road safety and productivity, benefitting both drivers and society as a whole.

## 5. Conclusion

Finally, this research aimed to accomplish two basic goals: first, to explore the influence of driving fatigue on the driving performance of Malaysian oil and petrol tanker drivers, and second, to define the precise kind of fatigue experienced by these drivers. Through comprehensive research and analysis, this study gave useful insights into the complex link between fatigue and driving performance in this environment.

According to the results, Malaysian oil and petrol tanker drivers do suffer from driving fatigue, with the consequences progressively mounting over time. Furthermore, the research highlighted the sort of fatigue that these drivers often experience, offering insight into the multidimensional nature of this problem. These findings are critical for our drivers’ well-being and safety, and they have far-reaching ramifications for the industry as a whole. Understanding the subtleties of driving fatigue and its impact allows us to work toward adopting effective methods and treatments to lessen its negative consequences, hence improving the safety and performance of Malaysian oil and petrol tanker drivers.

### 5.1. Theoretical implications

As a theoretical contribution, the study extended the body of knowledge regarding how driving fatigue impacts driving performance during actual driving among the drivers of Malaysian oil and gas tankers. This is one of the first studies using PVT in the Malaysian energy transportation industry to assess driving fatigue. The results clearly show how driving fatigue affects driving performance and what kind of fatigue drivers suffer.

### 5.2. Managerial implications

On the other hand, in terms of managerial contributions, this study provides empirical evidence to oil and gas transportation company managers. It then aids them in making decisions about the layout of drivers’ work schedules to prevent driving fatigue. Thus, it is recommended to change driver’s schedules and divide their 2 days as the weekend to be a day off every 3 days to improve the driver’s alertness effectively. Furthermore, train drivers in fatigue management to avoid fatigue and promote their culture regarding fatigue causes.

### 5.3. Limitations and future research

Despite its contributions, this study has many limitations that future research should consider. For instance, the study used only one device, which complicated the experiment, consumed a lot of time on data collection, and delayed the drivers’ time. Thus, future research can depend on developing an application drivers can download on their cell phones to monitor fatigue. Applications will allow the researcher to expand the sample and monitor fatigue for more time. In addition, this study measured drivers for only one shift. Therefore, it is recommended to redesign the future experiment to test each shift independently to compare the two shifts.

## Data availability statement

The raw data supporting the conclusions of this article will be made available by the authors, under reasonable reasons.

## Ethics statement

The studies involving human participants were reviewed and approved by the ethical committee of the Universiti Teknologi PETRONAS’s management and humanities department. The patients/participants provided their written informed consent to participate in this study.

## Author contributions

A-BA-M: writing—original draft, visualization, investigation, data collection, and data analysis. AI: visualization, supervision and quality control, and data collection. MA-Q: data analysis and writing—review and editing. NK: writing- review and editing. All authors contributed to the article and approved the submitted version.
